# Quantum teleportation of shared quantum secret in amplitude-damping channel

**DOI:** 10.1371/journal.pone.0334902

**Published:** 2025-11-10

**Authors:** Yimamujiang Aisan, Nueraminaimu Maihemuti, Jiayin Peng, Zhongwen Wang, Jiangang Tang

**Affiliations:** School of Mathematics and Statistics, Kashi University, Kashi, Xinjiang, China; Universite Cote d’Azur, FRANCE

## Abstract

We give the detailed processes for sharing a four-qubit pure entangled state as quantum channel in amplitude damping (AD) network channel via entanglement compensation.We propose a secure (2,2)-type quantum teleportation (QT) scheme based on this AD network channel, which allows 2-dimensional quantum information shared by 2 senders to be teleported to 2 receivers in such a way that after performing two Bell-state measurements by two senders, the original target state can be probabilistically reconstructed through introducing an auxiliary qubit and executing appropriate local unitary operations provided that all the receivers collaborate. We then extend it to the transmission of a 2-dimensional quantum secret state shared by *n* senders to *m* receivers (i.e., (*n*,*m*)-type QT of shared 2-dimensional quantum secret) from the perspectives of projective measurement, positive-operator-valued measurement (POVM) and generalized Bell-state measurement. Furthermore, we generalize the above (*n*,*m*)-type QT to the case of transmitting a shared *d*-dimensional quantum secret state. Our approach enables efficient and distributed quantum information relay, eliminating the need for a fully trusted central or intermediary node. The results show that our schemes achieve unit fidelity, though the success probabilities are less than 1. More interestingly, the QT protocol for high-dimensional quantum states exhibits a higher success probability than low-dimensional states under equivalent AD conditions.

## 1 Introduction

Since the proposal of QT in 1993 [1], it has been one of the most celebrated and extensively studied protocols for exploiting quantum entanglement [2]. This technique provides a way for two distant users to take advantage of the nonlocal correlations of an initially shared Einstein-Podolsky-Rosen state to teleport an arbitrary unknown single-qubit state from one site to another. QT is a unique phenomenon in quantum mechanics, without a counterpart in classical physics, and plays an important role in quantum information processing including secure quantum communication and quantum computing. Ever since Bennett et al. [1] first discovered that the information of an unknown qubit can be split into fragments and then recovered using classical information and quantum correlations, scholars have shown great interest in QT. On the other hand, some experiments soon demonstrated the QT of unknown single-qubit state with entangled photonic, atomic and ionic qubits [3–14]. Moreover, many theoretical protocols for transmitting an unknown quantum state through various quantum channels have been proposed [15–26]. Specifically, in a *d*-dimensional quantum system, if one has a resource of maximally entangled qudits and a measurement apparatus capable of distinguishing the *d*^2^ elements of the *d*-dimensional Bell basis, then an arbitrary multi-qudit state can be transmitted between two distant locations even without previous knowledge of it [16,18].

The QT protocol [1] enables precise transfer of quantum states between two locations, and its extension to multiple network nodes or end-users becomes critical for building scalable quantum communication infrastructures. Controlled teleportation, initially proposed in theoretical frameworks [27–30], has been experimentally validated [31–33], enabling directional distribution of quantum states from a single source to multiple receivers. This operational framework for multipartite quantum secret sharing (QSS) with enhanced security constraints [34–36] builds upon the foundational theories of Cleve et al. [37] and Gottesman [38]. In 1999, Cleve et al. [37] investigated the concept of quantum secret sharing, proposing threshold schemes and proving that (*k*,*n*) quantum secret sharing requires *n*<2*k*, providing a systematic construction method for such protocols. Later, Gottesman [38] introduced a variety of results on the theory of quantum secret sharing, establishing key principles for QSS, including the requirement for share sizes to match the secret’s dimension and the constraints imposed by the quantum no-cloning theorem.As QSS protocols advanced, these theoretical foundations were further extended. For example, Yuan et al. [39] proposed a three-party QSS scheme that allows for the deterministic recovery of arbitrary two-qutrit states through collaborative unitary operations, which was later generalized to multi-party systems. These developments align with Gottesman’s theoretical framework [38] regarding the dimensional requirements of shares and the design of access structures. Wei et al. [40] advanced the multi-party QSS of (n+1)-qudit states through single-qudit operations and measurements, while Wang et al. [41] extended this approach to multi-qudit states via dimensional scalability, the protocol is capable of distributing arbitrary multi-qubit states via collaborative control, and later generalized it through dimensional extensions to handle arbitrary unknown multi-qudit states within the same verification framework.Building on these architectures, a hierarchical quantum computing paradigm with centralized governance has been experimentally demonstrated [42], featuring orchestrated control across distributed quantum processors. In parallel, specialized quantum communication protocols [43,44] have been developed to underpin systematic construction of scalable quantum network infrastructures with adaptive resource allocation capabilities. However, none of the above QT schemes allows us to perform quantum teleportation between multiple senders and multiple receivers. Especially, they lack the ability to directly transmit shared or split quantum information among multiple parties to other agents without concentrating the information in the location of single or subparties, which has led to the requirement of fully trusted central or intermediate nodes in the design of quantum communication networks [45,46]. Recently, Lee et al. [47] presented and experimentally demonstrated a secure QT between multiple senders and receivers, i.e., it can transmit quantum information shared by an arbitrary number of senders to another arbitrary number of receivers, where neither any single nor subparties of senders and receivers can fully access the secret quantum information. This scheme allows us to relay quantum information over a network in an efficient and distributed manner without requiring fully trusted central or intermediate nodes. Subsequently, Li et al. [48] replaced the quantum network channel in Ref [47] with a partially entangled GHZ state to achieve the quantum teleportation of shared quantum secret. In 2024, Peng et al. [49] generalized the results of Lee et al. [47] to the case of teleporting a shared high-dimensional quantum secret. However, most QT schemes focus solely on ideal, noise-free quantum channels, neglecting the impact of channel noise on their performance in real quantum communication.In fact, any open quantum system is inevitably affected by environmental noise during its interaction with the environment, leading to decoherence [50].This implies that whether quantum resources are distributed in a maximally entangled state or a purely entangled state, channel noise inevitably converts the pure state into a mixed entangled state and can even cause entanglement sudden death [51]. Environmental noise leads to various types of decoherence, with AD being a typical mechanism. AD not only represents decoherence due to the dissipative process from the interaction between the quantum system and its environment, but also captures many crucial noise features within the quantum system [52,53]. So far, many scholars have also conducted in-depth research on the QT protocol through AD channel [54–56]. The research shows that distributing quantum resources in an AD noisy channel can reduce the fidelity of the teleported quantum state in the QT scheme. To improve fidelity in non-ideal environments, researchers have employed quantum weak measurement techniques to enhance the fidelity of the teleported state in AD channels [57–59].However, after quantum weak measurement and its inverse measurement, the fidelity of the transmitted state in the AD channel is still unable to reach 1. More importantly, as the attenuation rate of the decoherent channel and weak measurement strength increase in the AD channel, the success probability of the corresponding QT scheme rapidly decreases to zero. Very recently, Hu et al. [60] presented two multiparty schemes in AD channel with unit fidelity that, respectively share an arbitrary unknown single-qubit state and single-qutrit state. That is, they considered the exact sharing of single-particle states in two-level and three-level quantum systems based on AD channels.

In this paper, we integrate the core ideas of Refs. [47] and [60], but this is not a simple superposition of the two; rather, it is a systematic scheme constructed through in-depth integration and targeted innovation. Specifically, we innovatively combine the many-to-many interaction framework of the former with the noise robustness technology of the latter, expanding the research scenario to the new direction of “QT of shared quantum secrets in AD channel". We propose a conclusive (2,2)-type QT scheme to realize the transmission of shared 2-dimensional quantum secret states between two senders and two receivers. The scheme consists of two key stages: in the first stage, stable sharing of a 2-dimensional four-qubit pure entangled state between senders and receivers is established in the amplitude-damping channel via optimized entanglement compensation technology; in the second stage, after the senders complete two Bell-state measurements, the receivers accurately reconstruct the original target state by introducing an ancillary qubit and executing local unitary operations adapted to multi-party collaboration. Building on this, we generalize the above scheme to the (*n*,*m*)-type quantum teleportation scenario, which supports the transmission of 2-dimensional quantum information shared by *n* senders to *m* receivers. Innovatively, we achieve this extension through three measurement methods—projective measurement, positive operator-valued measurement, and generalized Bell-state measurement—breaking through the limitation of single measurement mode in existing schemes. Finally, we further extend the (*n*,*m*)-type teleportation of 2-dimensional shared quantum information to the scenario of *d*-dimensional shared quantum information. Research shows that both the teleportation of shared 2-dimensional and *d*-dimensional quantum secrets can reach the target receivers with unit fidelity, with the corresponding trade-off that the success probabilities of both schemes are less than 1.

The organization of this article is as follows: Sect 2 proposes a conclusive and secure (2,2)-type QT scheme between two senders and two receivers in an AD channel. Its generalized version, which describes the transmission of 2-dimensional quantum information shared by *n* senders to *m* receivers in an AD channel, is presented in Sect 3. Sect 4 extends the discussion in Sect 3, generalizing the transmission of 2-dimensional shared quantum information to the case of *d*-dimensional shared quantum information. Finally, Sect 5 provides the discussion and conclusion.

## 2 (2,2)-Type QT of shared 2-dimensional quantum secret in AD channel

Quantum noise is an unavoidable phenomenon in quantum teleportation. Particles that make up the entangled channel suffer from quantum noise during the distribution among multiple participants. Hence, the quantum state that forms the channel changes from pure state to mixed state and it affects the fidelity of the output state in quantum teleportation schemes seriously. An important class of noise related to losses in quantum systems is known as AD noise, which has been used to model various phenomena including idle errors in quantum computing, energy dissipation, spontaneous photon emission, and attenuation, among others in two level systems. For a single qubit in a 2-dimensional quantum system, the Kraus operators for AD noise are defined as:

$$K_{0}=\left(\begin{array}{cc} 1 & 0\\ 0 & \sqrt{1-\gamma } \end{array}\right),\;K_{1}=\left(\begin{array}{cc} 0 & \sqrt{\gamma }\\ 0 & 0 \end{array}\right),$$ (1)

where 0≤γ≤1 represents the amplitude damping strength.

In this section, we will explain in detail the (2,2)-type QT scheme that supposes a quantum secret in $|S_{2}\rangle =\alpha |0_{L}\rangle \;+\;\beta |1_{L}\rangle$ with logical basis $|0_{L}\rangle$ and $|1_{L}\rangle$, which is shared by separated 2 parties in quantum network through a splitting protocol [27–30]. We utilize a pure Greenberger-Horne-Zeilinger (GHZ) state to encode both the network qubits and the logical qubits. For a 2-qubit system, the specific form of this GHZ state is $|\text{GHZ}\rangle =\frac{1}{\sqrt{2}}(|00\rangle \;+\;|11\rangle )$. Alice and Bob, the two senders,attempt to transmit the secret to Charlie and David,the two receivers connected within the network, using an AD channel. None of the participants are fully trusted in this scenario, ensuring that neither the senders nor the receivers can access the secret individually throughout the entire process.The secret state designated for teleportation is represented by $|S_{2}\rangle =(\alpha |00\rangle \;+\;\beta |11\rangle {)}_{s_{1}s_{2}}$, where *α* and *β* are probability amplitudes satisfying $|\alpha {|}^{2}\;+\;|\beta {|}^{2}=1$,and *s*_1_ and *s*_2_ are qubits held by Alice and Bob respectively. Upon transmission, the receivers obtain the shared secret state $|R_{2}\rangle =(\alpha |00\rangle \;+\;\beta |11\rangle {)}_{r_{1}r_{2}}$, with *r*_1_ and *r*_2_ assigned to Charlie and David respectively.

To complete the QT task, we need to go through the following stages.

**Stage 1** Preparation of quantum channel.

(a1) Alice generates a Bell state $|{ℬ}_{00}\rangle =\frac{1}{\sqrt{2}}(|00\rangle +|11\rangle {)}_{AA_{1}}$. Afterwards, she sends the qubit *A*_1_ to Bob through an amplitude damping channel.

(a2) After Bob receives the qubit *A*_1_, he first introduces an ancillary qubit *B* in the initial state $|0\rangle_{B}$, and then sends qubit pair (*A*_1_,*B*) into a CNOT gate, where qubits *A*_1_ and *B* serve as the control and target particle, respectively. The quantum operator corresponding to the CNOT gate is defined as:

$$CNOT=|00\rangle_{12}\langle 00|+|01\rangle_{12}\langle 01|+|11\rangle_{12}\langle 10|+|10\rangle_{12}\langle 11|,$$ (2)

where subscripts 1 and 2, respectively represent the control qubit and target qubit. After that, Bob delivers qubit *A*_1_ to Charlie via the amplitude damped channel.

(a3) After Charlie receives the qubit *A*_1_, he also introduces an auxiliary qubit *C* in the initial state $|0\rangle_{C}$ and performs a CNOT gate on qubit pair (*A*_1_,*C*), where qubit *A*_1_ acts as the control particle and qubits *C* as target particle. Then, he transmits qubit *A*_1_ to David through the amplitude damped channel.

(a4) When David receives the qubit *A*_1_, he introduces an auxiliary qubit *D* in the initial state $|0\rangle_{D}$ and performs a CNOT gate on qubit pair (*A*_1_,*D*), where qubit *A*_1_ acts as the control particle and qubit *D* as target particle. Then, he transmits qubit *A*_1_ to Alice through the amplitude damped channel.

(a5) After receiving qubit *A*_1_, Alice initially performs the CNOT operation on the qubit pair (*A*,*A*_1_), where qubit *A* acts as the control particle and qubit *A*_1_ as the target particle. She then conducts a single-qubit projective measurement on her qubit *A*_1_ in the *Z*-basis {|0⟩,|1⟩}. If Alice’s measurement outcome is $|0\rangle_{A_{1}}$, the scheme proceeds to Step 1 in Stage 2. Otherwise, the scheme terminates and restarts from scratch until Alice obtains a successful measurement outcome $|0\rangle_{A_{1}}$.

From the above (a5), if Alice gains the measurement results $|0\rangle_{A_{1}}$ successfully,the collective state of the qubits (*A*,*A*_1_,*B*,*C*,*D*) collapses into a purely entangled state:

$$|ℋ\rangle_{ABCD}=\frac{1}{\sqrt{1+(1-\gamma {)}^{4}}}[|0000\rangle +(1-\gamma {)}^{2}|1111\rangle {]}_{ABCD}$$ (3)

and the detailed proof of Eq (3) can be found in S1 Appendix. That is, Alice, Bob and Charlie have successfully shared the four-qudit quantum state $|ℋ\rangle_{ABCD}$.

**Stage 2** (2,2)-type QT of shared 2-dimensional information.

The initial composite system consists of the shared secret and the shared pure entangled state.The composite state is expressed as:

$$|𝒯\rangle =|S_{2}\rangle ⊗|ℋ\rangle_{ABCD}\;=\frac{1}{\sqrt{1+(1-\gamma {)}^{4}}}(\alpha |00\rangle +\beta |11\rangle {)}_{s_{1}s_{2}}[|0000\rangle +(1-\gamma {)}^{2}|1111\rangle {]}_{ABCD}\;=\frac{1}{\sqrt{2+2(1-\gamma {)}^{4}}}\{|\Phi_{2}^{+}\rangle_{s_{1}s_{2}AB}[\alpha |00\rangle_{r_{1}r_{2}}+(1-\gamma {)}^{2}\beta |11\rangle_{r_{1}r_{2}}]\;\;+|\Phi_{2}^{-}\rangle_{s_{1}s_{2}AB}[\alpha |00\rangle_{r_{1}r_{2}}-(1-\gamma {)}^{2}\beta |11\rangle_{r_{1}r_{2}}]\;\;+|\Psi_{2}^{+}\rangle_{s_{1}s_{2}AB}[(1-\gamma {)}^{2}\alpha |11\rangle_{r_{1}r_{2}}+\beta |00\rangle_{r_{1}r_{2}}]\;\;+|\Psi_{2}^{-}\rangle_{s_{1}s_{2}AB}[(1-\gamma {)}^{2}\alpha |11\rangle_{r_{1}r_{2}}-\beta |00\rangle_{r_{1}r_{2}}],$$ (4)

with

$$|\Phi_{2}^{\pm }\rangle_{s_{1}s_{2}AB}=\frac{1}{\sqrt{2}}(|0000\rangle \pm |1111\rangle {)}_{s_{1}s_{2}AB},\;|\Psi_{2}^{\pm }\rangle_{s_{1}s_{2}AB}=\frac{1}{\sqrt{2}}(|0011\rangle \pm |1100\rangle {)}_{s_{1}s_{2}AB}.$$ (5)

***Step* 1 in Stage 2** The measurements of senders.

By adjusting the order of the four qubits in Eq (4) from $\left(s_{1},s_{2},A,B\right)$ to $\left(s_{1},A,s_{2},B\right)$, we obtain the following (with subscripts omitted for brevity):

$$|\Phi_{2}^{+}\rangle =\frac{1}{\sqrt{2}}(|\phi^{+}\rangle |\phi^{+}\rangle +|\phi^{-}\rangle |\phi^{-}\rangle ),\;|\Phi_{2}^{-}\rangle =\frac{1}{\sqrt{2}}(|\phi^{+}\rangle |\phi^{-}\rangle +|\phi^{-}\rangle |\phi^{+}\rangle ),\;|\Psi_{2}^{+}\rangle =\frac{1}{\sqrt{2}}(|\psi^{+}\rangle |\psi^{+}\rangle +|\psi^{-}\rangle |\psi^{-}\rangle ),\;|\Psi_{2}^{-}\rangle =\frac{1}{\sqrt{2}}(|\psi^{+}\rangle |\psi^{-}\rangle +|\psi^{-}\rangle |\psi^{+}\rangle ),\;$$ (6)

where the Bell states are defined as

$$|\phi^{\pm }\rangle =\frac{1}{\sqrt{2}}(|00\rangle \pm |11\rangle ),\;|\psi^{\pm }\rangle =\frac{1}{\sqrt{2}}(|01\rangle \pm |10\rangle ).$$ (7)

The senders Alice and Bob carry out the Bell-state measurements on the qubit pairs (*s*_1_,*A*) and (*s*_2_,*B*), respectively. After conducting two Bell-state measurements, Alice and Bob transmit the measurement outcomes to the receivers Charlie and David through classical communication channels. The measurement outcome for Alice or Bob is denoted as $z_{j}x_{j}$, which corresponds to *s*_*j*_. The classical bit pairs {00,10,01,11} are mapped to the Bell states $\left\{|\phi^{+}\rangle ,|\phi^{-}\rangle ,|\psi^{+}\rangle ,|\psi^{-}\rangle \right\}$.

***Step* 2 in Stage 2** The receivers’ reconstructions of the target state.

(c1) If the measurement result is $|\Phi_{2}^{+}\rangle$ or $|\Phi_{2}^{-}\rangle$ from Alice and Bob, each of Charlie and David performs the local Pauli operation $\sigma^{Z}$, where $Z=(z_{1}+z_{2})mod2$, and the collapsed state of Charlie and David will be changed into

$$|R_{2}^{\prime }\rangle =\frac{1}{\sqrt{|\alpha {|}^{2}+(1-\gamma {)}^{4}|\beta {|}^{2}}}[\alpha |00\rangle +(1-\gamma {)}^{2}\beta |11\rangle {]}_{r_{1}r_{2}}.$$ (8)

To accurately reconstruct the initial state with unity fidelity, it is necessary to introduce an auxiliary qubit in the state $|0\rangle_{au}$. This auxiliary qubit will play a crucial role in ensuring the fidelity of the state reconstruction.Owing to the symmetry of the qubits in state $|R_{2}^{\prime }\rangle$, the auxiliary qubit can be held by any one of the receivers, Charlie and David. Without loss of generality, we can assume that David holds the auxiliary qubit and subsequently performs a collective unitary transformation. This transformation will be applied to the combined system in order to reconstruct the initial state.

$$U_{1}=\left(\begin{array}{cccc} (1-\gamma {)}^{2} & \sqrt{1-(1-\gamma {)}^{4}} & 0 & 0\\ -\sqrt{1-(1-\gamma {)}^{4}} & (1-\gamma {)}^{2} & 0 & 0\\ 0 & 0 & 1 & 0\\ 0 & 0 & 0 & 1 \end{array}\right)$$ (9)

under the basis $\left\{|0\rangle_{D}|0\rangle_{au},|0\rangle_{D}|1\rangle_{au},|1\rangle_{D}|0\rangle_{au},|1\rangle_{D}|1\rangle_{au}\right\}$. It transforms the product state $|R_{2}^{\prime }\rangle ⊗|0\rangle_{au}$ to

$$(I⊗U_{1})(|R_{2}^{\prime }\rangle ⊗|0\rangle_{au})=\frac{(1-\gamma {)}^{2}}{\sqrt{|\alpha {|}^{2}+(1-\gamma {)}^{4}|\beta {|}^{2}}}(\alpha |00\rangle +\beta |11\rangle {)}_{CD}⊗|0\rangle_{au}\;\;+\frac{\alpha \sqrt{1-(1-\gamma {)}^{4}}}{\sqrt{|\alpha {|}^{2}+(1-\gamma {)}^{4}|\beta {|}^{2}}}|00\rangle_{CD}⊗|1\rangle_{au}.$$ (10)

David then performs a single-qubit projective measurement on the auxiliary qubit in the *Z*-basis {|0⟩,|1⟩}. If the result is $|0\rangle_{au}$, teleportation succeeds with fidelity 1. However, if the result is $|1\rangle_{au}$, teleportation fails and no information about the target state is obtained. The optimal success probability is given by $\left(1-\gamma {)}^{4}/[1+(1-\gamma {)}^{4}\right]$, where “optimal" refers to the inclusion of the auxiliary qubit.

(c2) If the senders’ measurement result is $|\Psi_{2}^{+}\rangle$ or $|\Psi_{2}^{-}\rangle$, one of the receivers, either Charlie or David, applies the local Pauli operation $\sigma^{X}\sigma^{Z}$, where $X=(x_{1}+x_{1})\;\mathrm{mod}\;2$, and the other receiver applies the Pauli operation $\sigma^{X}$. This process will cause the collapsed state owned by the receivers to become the desired state:

$$|R_{2}^{\prime \prime }\rangle =\frac{1}{\sqrt{(1-\gamma {)}^{4}|\alpha {|}^{2}+|\beta {|}^{2}}}[(1-\gamma {)}^{2}\alpha |00\rangle +\beta |11\rangle {]}_{r_{1}r_{2}}.$$ (11)

Similarly, an auxiliary qubit *D* in the initial state $|0\rangle_{au}$ is introduced at the location of the receiver David, and the corresponding collective unitary transformation under the basis $\left\{|0\rangle_{D}|0\rangle_{au},|0\rangle_{D}|1\rangle_{au},|1\rangle_{D}|0\rangle_{au},|1\rangle_{D}|1\rangle_{au}\right\}$ is

$$U_{2}=\left(\begin{array}{cccc} 1 & 0 & 0 & 0\\ 0 & 1 & 0 & 0\\ 0 & 0 & (1-\gamma {)}^{2} & \sqrt{1-(1-\gamma {)}^{4}}\\ 0 & 0 & -\sqrt{1-(1-\gamma {)}^{4}} & (1-\gamma {)}^{2} \end{array}\right),$$ (12)

which will transform the product state $|R_{2}^{\prime \prime }\rangle ⊗|0\rangle_{au}$ to

$$(I⊗U_{2})(|R_{2}^{\prime \prime }\rangle ⊗|0\rangle_{au})=\frac{(1-\gamma {)}^{2}}{\sqrt{(1-\gamma {)}^{4}|\alpha {|}^{2}+|\beta {|}^{2}}}(\alpha |00\rangle +\beta |11\rangle {)}_{CD}⊗|0\rangle_{au}\;\;+\frac{\beta \sqrt{1-(1-\gamma {)}^{4}}}{\sqrt{(1-\gamma {)}^{4}|\alpha {|}^{2}+|\beta {|}^{2}}}|11\rangle_{CD}⊗|1\rangle_{au}.$$ (13)

Next, David performs a single-qubit measurement in the computational basis on the auxiliary qubit. The target state can only be reconstructed if the result is $|0\rangle_{au}$, with a probability of $\left(1-\gamma {)}^{4}/[1+(1-\gamma {)}^{4}\right]$. By considering both contributions, the optimal probability of successful teleportation is $2(1-\gamma {)}^{4}/[1+(1-\gamma {)}^{4}]$.

Interestingly, when γ=0(i.e., amplitude damping noise vanishes), $|ℋ\rangle_{ABCD}$ becomes a maximally entangled four-qubit GHZ state, and $U_{1}=U_{2}\equiv I$, meaning David does not need to do anything on the corresponding operations.Thus, the above scheme reduces to the standard protocol for quantum teleportation of shared quantum secret Ref [47]. In this sense, our scheme is a generalization of that in Ref [47].

## 3 (*n*,*m*)-Type QT of shared 2-dimensional quantum secret in AD channel

In the previous section, we describe in detail the (2,2)-type quantum teleportation of shared 2-dimensional quantum secret in amplitude damped channel. Now let’s generalize this (2,2)-type scheme to that of (*n*,*m*)-type, where *n* and *m* are any natural numbers greater than 2. Suppose a quantum secret in $|S_{n}\rangle =\alpha {⊗}_{j=1}^{n}|0\rangle_{s_{j}}+\beta {⊗}_{j=1}^{n}|1\rangle_{s_{j}}$ was previously shared among *n* separated parties,denoted as $\left\{s_{1},s_{2},\ldots ,s_{n}\right\}$, in the quantum network via a splitting protocol.The senders, a group $\left\{s_{1},s_{2},\ldots ,s_{n}\right\}$ of *n* parties, attempt to teleport the quantum secret to the receivers, another group $\left\{r_{1},r_{2},\ldots ,r_{m}\right\}$ of *m* parties, connected through the network. Of course, the secret state obtained at the receivers’ hands can be reconstructed as $|R_{m}\rangle =\alpha {⊗}_{j=1}^{m}|0\rangle_{r_{j}}+\beta {⊗}_{j=1}^{m}|1\rangle_{r_{j}}$. None of the participants is fully trusted, so no individual or subgroup of senders or receivers is allowed to access the quantum secret during the entire process.

Using a similar quantum channel preparation method as in Stage 1 of Sect 2, the senders and receivers can share an (n+m)-qubit pure entangled state, written as

$$|ℋ\rangle_{n+m}=\frac{1}{\sqrt{1+(1-\gamma {)}^{n+m}}}[{⊗}_{i=1}^{n}|0\rangle_{s_{i}^{\prime }}{⊗}_{j=1}^{m}|0\rangle_{r_{j}}\;\;+\sqrt{(1-\gamma {)}^{n+m}}{⊗}_{i=1}^{n}|1\rangle_{s_{i}^{\prime }}{⊗}_{j=1}^{m}|1\rangle_{r_{j}}].$$ (14)

where $s_{i}^{\prime }(i=1,2,\ldots ,n)$ are auxiliary qubits held by the senders to construct the quantum channel. Thus, by using the Bell states as shown in Eq (7), the initial composite system $|S_{n}\rangle |ℋ\rangle_{n+m}$ can be written as

$$|S_{n}\rangle |ℋ\rangle_{n+m}\;=\frac{1}{\sqrt{2+2(1-\gamma {)}^{n+m}}}\{|\Phi_{n}^{+}\rangle_{s_{1}\cdots s_{n}s_{1}^{\prime }\cdots s_{n}^{\prime }}[\alpha {⊗}_{j=1}^{m}|0\rangle_{r_{j}}\;\;+\sqrt{(1-\gamma {)}^{n+m}}\beta {⊗}_{j=1}^{m}|1\rangle_{r_{j}}]\;\;+|\Phi_{n}^{-}\rangle_{s_{1}\cdots s_{n}s_{1}^{\prime }\cdots s_{n}^{\prime }}[\alpha {⊗}_{j=1}^{m}|0\rangle_{r_{j}}-\sqrt{(1-\gamma {)}^{n+m}}\beta {⊗}_{j=1}^{m}|1\rangle_{r_{j}}]\;\;+|\Psi_{n}^{+}\rangle_{s_{1}\cdots s_{n}s_{1}^{\prime }\cdots s_{n}^{\prime }}[\sqrt{(1-\gamma {)}^{n+m}}\alpha {⊗}_{j=1}^{m}|1\rangle_{r_{j}}+\beta {⊗}_{j=1}^{m}|0\rangle_{r_{j}}]\;\;+|\Psi_{n}^{-}\rangle_{s_{1}\cdots s_{n}s_{1}^{\prime }\cdots s_{n}^{\prime }}[\sqrt{(1-\gamma {)}^{n+m}}\alpha {⊗}_{j=1}^{m}|1\rangle_{r_{j}}-\beta {⊗}_{j=1}^{m}|0\rangle_{r_{j}}]\}\;$$ (15)

where

$$|\Phi_{n}^{\pm }\rangle_{s_{1}\cdots s_{n}s_{1}^{\prime }\cdots s_{n}^{\prime }}=\frac{1}{\sqrt{2}}({⊗}_{l=1}^{n}|0\rangle_{s_{l}}{⊗}_{l=1}^{n}|0\rangle_{s_{l}^{\prime }}\pm {⊗}_{l=1}^{n}|1\rangle_{s_{l}}{⊗}_{l=1}^{n}|1\rangle_{s_{l}^{\prime }}),\;|\Psi_{n}^{\pm }\rangle_{s_{1}\cdots s_{n}s_{1}^{\prime }\cdots s_{n}^{\prime }}=\frac{1}{\sqrt{2}}({⊗}_{l=1}^{n}|0\rangle_{s_{l}}{⊗}_{l=1}^{n}|1\rangle_{s_{l}^{\prime }}\pm {⊗}_{l=1}^{n}|1\rangle_{s_{l}}{⊗}_{l=1}^{n}|0\rangle_{s_{l}^{\prime }}).\;$$ (16)

To complete this quantum teleportation task, we need to adjust the order of 2*n* qubits from $\left(s_{1},s_{2},\cdots ,s_{n},s_{1}^{\prime },s_{2}^{\prime },\cdots ,s_{n}^{\prime }\right)$ to $\left(s_{1},s_{1}^{\prime },s_{2},s_{2}^{\prime },\cdots ,s_{n},s_{n}^{\prime }\right)$, resulting in the following state (subscripts omitted for brevity):

$$|\Phi_{n}^{+}\rangle =\frac{1}{\sqrt{2^{n-1}}}\sum_{0\leq k=0mod2\leq n}N[|\phi^{-}\rangle^{⊗k}|\phi^{+}\rangle^{⊗(n-k)}],\;|\Phi_{n}^{-}\rangle =\frac{1}{\sqrt{2^{n-1}}}\sum_{0\leq k=1mod2\leq n}N[|\phi^{-}\rangle^{⊗k}|\phi^{+}\rangle^{⊗(n-k)}],\;|\Psi_{n}^{+}\rangle =\frac{1}{\sqrt{2^{n-1}}}\sum_{0\leq k=0mod2\leq n}N[|\psi^{-}\rangle^{⊗k}|\psi^{+}\rangle^{⊗(n-k)}],\;|\Psi_{n}^{-}\rangle =\frac{1}{\sqrt{2^{n-1}}}\sum_{0\leq k=1mod2\leq n}N[|\psi^{-}\rangle^{⊗k}|\psi^{+}\rangle^{⊗(n-k)}],\;$$ (17)

where N[·] represents the sum of all possible permutations. For example,

$$N[|\phi^{-}\rangle |\phi^{+}\rangle^{⊗3}]=|\phi^{-}\rangle |\phi^{+}\rangle |\phi^{+}\rangle |\phi^{+}\rangle +|\phi^{+}\rangle |\phi^{-}\rangle |\phi^{+}\rangle |\phi^{+}\rangle \;\;+|\phi^{+}\rangle |\phi^{+}\rangle |\phi^{-}\rangle |\phi^{+}\rangle +|\phi^{+}\rangle |\phi^{+}\rangle |\phi^{+}\rangle |\phi^{-}\rangle .$$ (18)

Each sender *s*_*j*_ performs a standard Bell-state measurement on their two qubits *s*_*j*_ and $s_{j}^{\prime }$, then informs the receivers of the result through classical channels. After performing *n* Bell-state measurements, the state of the qubits $r_{1},r_{2},\ldots ,r_{m}$ will collapse into one of the following states:

$$\alpha {⊗}_{j=1}^{m}|0\rangle_{r_{j}}\pm \sqrt{(1-\gamma {)}^{n+m}}\beta {⊗}_{j=1}^{m}|1\rangle_{r_{j}},\;\sqrt{(1-\gamma {)}^{n+m}}\alpha {⊗}_{j=1}^{m}|1\rangle_{r_{j}}\pm \beta {⊗}_{j=1}^{m}|0\rangle_{r_{j}}.$$ (19)

Define the measurement result of the sender *s*_*j*_ as $z_{j}x_{j}$, with the classical bits {00,10,01,11} corresponding to the Bell states $\left\{|\phi^{+}\rangle ,|\phi^{-}\rangle ,|\psi^{+}\rangle ,|\psi^{-}\rangle \right\}$. When the measurement result is $|\Phi^{+}\rangle$ or $|\Phi^{-}\rangle$, each receiver *r*_*j*_ applies the local Pauli operator $\sigma_{z}^{Z}$ ($Z=(\sum_{j=1}^{n}z_{j})\;\mathrm{mod}\;2$) on their qubit *r*_*j*_, and the collapsed state becomes

$$|R^{\prime }\rangle =\frac{1}{\sqrt{|\alpha {|}^{2}+(1-\gamma {)}^{n+m}|\beta {|}^{2}}}[\alpha {⊗}_{j=1}^{m}|0\rangle_{r_{j}}+\sqrt{(1-\gamma {)}^{n+m}}\beta {⊗}_{j=1}^{m}|1\rangle_{r_{j}}].$$ (20)

If the measurement result is $|\Psi^{+}\rangle$ or $|\Psi^{-}\rangle$, one of the receivers applies the local Pauli operator $\sigma_{x}^{X}\sigma_{z}^{Z}$ ($X=(\Sigma_{j=1}^{n}x_{j})mod2$), while the other receivers apply the operator $\sigma_{x}^{X}$. This will result in the collapsed state becoming

$$|R^{\prime \prime }\rangle =\frac{1}{\sqrt{(1-\gamma {)}^{n+m}|\alpha {|}^{2}+|\beta {|}^{2}}}[\sqrt{(1-\gamma {)}^{n+m}}\alpha {⊗}_{j=1}^{m}|0\rangle_{r_{j}}+\beta {⊗}_{j=1}^{m}|1\rangle_{r_{j}}].$$ (21)

### 3.1 (*n*,*m*)-Type QT based on projective measurement

Apparently, the states $|R^{\prime }\rangle$ and $|R^{\prime \prime }\rangle$ have not yet been reconstructed by the receivers as the intended target states. To achieve the restoration of the initial state with perfect fidelity, it is necessary to introduce an auxiliary qubit initialized in the state $|0\rangle_{au}$. First, consider the situation where the measurement outcome is either $|\Phi^{+}\rangle$ or $|\Phi^{-}\rangle$. Given the symmetry of the state $|R^{\prime }\rangle$, any receiver can possess the ancillary qubit. Without loss of generality, we can assume that the final receiver *r*_*m*_ holds the ancillary qubit and subsequently performs a unitary transformation.

$$U_{R^{\prime }}=\left(\begin{array}{cccc} \sqrt{(1-\gamma {)}^{n+m}} & \sqrt{1-(1-\gamma {)}^{n+m}} & 0 & 0\\ -\sqrt{1-(1-\gamma {)}^{n+m}} & \sqrt{(1-\gamma {)}^{n+m}} & 0 & 0\\ 0 & 0 & 1 & 0\\ 0 & 0 & 0 & 1 \end{array}\right)$$ (22)

under the basis $\left\{|0\rangle_{r_{m}}|0\rangle_{au},|0\rangle_{r_{m}}|1\rangle_{au},|1\rangle_{r_{m}}|0\rangle_{au},|1\rangle_{r_{m}}|1\rangle_{au}\right\}$. This transformation converts the product state $|R^{\prime }\rangle ⊗|0\rangle_{au}$ to

$$(I⊗U_{R^{\prime }})(|R^{\prime }\rangle ⊗|0\rangle_{au})=\frac{\sqrt{(1-\gamma {)}^{n+m}}}{\sqrt{|\alpha {|}^{2}+(1-\gamma {)}^{n+m}|\beta {|}^{2}}}|R_{m}\rangle ⊗|0\rangle_{au}\;\;+\frac{\alpha \sqrt{1-(1-r{)}^{n+m}}}{\sqrt{|\alpha {|}^{2}+(1-\gamma {)}^{n+m}|\beta {|}^{2}}}{⊗}_{j=1}^{m}|0\rangle_{r_{j}}⊗|1\rangle_{au}.$$ (23)

The receiver *r*_*m*_ then performs a single-qubit projective measurement on the auxiliary qubit in the *Z*-basis {|0⟩,|1⟩}. If the result is $|0\rangle_{au}$, the teleportation is successfully completed with unity fidelity. However, if the result is $|1\rangle_{au}$, the teleportation fails, and no information about the target state is obtained. The probability of success is $\left(1-\gamma {)}^{n+m}/[1+(1-\gamma {)}^{n+m}\right]$.

When the measurement result is $|\Psi^{+}\rangle$ or $|\Psi^{-}\rangle$, the receiver *r*_*m*_ similarly applies the corresponding collective unitary transformation.

$$U_{R^{\prime \prime }}=\left(\begin{array}{cccc} 1 & 0 & 0 & 0\\ 0 & 1 & 0 & 0\\ 0 & 0 & \sqrt{(1-\gamma {)}^{n+m}} & \sqrt{1-(1-\gamma {)}^{n+m}}\\ 0 & 0 & -\sqrt{1-(1-\gamma {)}^{n+m}} & \sqrt{(1-\gamma {)}^{n+m}} \end{array}\right)$$ (24)

under the basis $\left\{|0\rangle_{r_{m}}|0\rangle_{au},|0\rangle_{r_{m}}|1\rangle_{au},|1\rangle_{r_{m}}|0\rangle_{au},|1\rangle_{r_{m}}|1\rangle_{au}\right\}$. This transformation will change the product state $|R^{\prime \prime }\rangle ⊗|0\rangle_{au}$ to

$$(I⊗U_{R^{\prime \prime }})(|R^{\prime \prime }\rangle ⊗|0\rangle_{au})=\frac{\sqrt{(1-\gamma {)}^{n+m}}}{\sqrt{(1-\gamma {)}^{n+m}|\alpha {|}^{2}+|\beta {|}^{2}}}|R_{m}\rangle ⊗|0\rangle_{au}\;\;+\frac{\beta \sqrt{1-(1-r{)}^{n+m}}}{\sqrt{(1-\gamma {)}^{n+m}|\alpha {|}^{2}+|\beta {|}^{2}}}{⊗}_{j=1}^{m}|1\rangle_{r_{j}}⊗|1\rangle_{au}.$$ (25)

Next, the receiver *r*_*m*_ performs a single-qubit projective measurement on the auxiliary qubit in the basis {|0⟩,|1⟩}. Only if the result is $|0\rangle_{au}$, the target state can be reconstructed with probability $\left(1-\gamma {)}^{n+m}/[1+(1-\gamma {)}^{n+m}\right]$. Adding both contributions together, the optimal probability of successful teleportation is obtained as $2(1-\gamma {)}^{n+m}/[1+(1-\gamma {)}^{n+m}]$.

It is worth mentioning that when γ=0, |ℋ⟩ is a maximally entangled channel, and the success probability is 1, which means that this scheme is a generalization of that in Ref [47].

### 3.2 (*n*,*m*)-Type QT based on positive operator-valued measurement

Let us only consider Eq (20), as the discussion on Eq (21) yields the same result. After introducing an auxiliary qubit au with the original state $|0\rangle_{A}$ by the last receiver *r*_*m*_, he or she implements a controlled-NOT (CNOT) gate on qubits *r*_*m*_ and *A*, where *r*_*m*_ works as the control qubit and *A* as the target qubit. It will transform the product state $|R^{\prime }\rangle ⊗|0\rangle_{A}$ to

$$\frac{1}{\sqrt{|\alpha {|}^{2}+(1-\gamma {)}^{n+m}|\beta {|}^{2}}}[\alpha {⊗}_{j=1}^{m}|0\rangle_{r_{j}}|0\rangle_{A}\;\;+\sqrt{(1-\gamma {)}^{n+m}}\beta {⊗}_{j=1}^{m}|1\rangle_{r_{j}}|1\rangle_{A}]\;=\frac{1}{2\sqrt{|\alpha {|}^{2}+(1-\gamma {)}^{n+m}|\beta {|}^{2}}}(|E\rangle_{m}⊗|F\rangle_{A}+|G\rangle_{m}⊗|H\rangle_{A}),$$ (26)

where

$$|E\rangle_{m}=\alpha {⊗}_{j=1}^{m}|0\rangle_{r_{j}}+\beta {⊗}_{j=1}^{m}|1\rangle_{r_{j}},\;|F\rangle_{A}=|0\rangle_{A}+\sqrt{(1-\gamma {)}^{n+m}}|1\rangle_{A},\;|G\rangle_{m}=\alpha {⊗}_{j=1}^{m}|0\rangle_{r_{j}}-\beta {⊗}_{j=1}^{m}|1\rangle_{r_{j}},\;|H\rangle_{A}=|0\rangle_{A}-\sqrt{(1-\gamma {)}^{n+m}}|1\rangle_{A}.$$ (27)

To determine $|E\rangle_{m}$ and $|G\rangle_{m}$, the receiver *r*_*m*_ must perform a POVM on the auxiliary qubit *A*. The POVM should be structured as follows:

$$O_{1}=\frac{1}{\mu }|M_{1}\rangle \langle M_{1}|,\;O_{2}=\frac{1}{\mu }|M_{2}\rangle \langle M_{2}|,\;O_{3}=I-\frac{1}{\mu }\sum_{j=1}^{2}|M_{j}\rangle \langle M_{j}|,$$ (28)

in this case,*I* denotes the identity operator,and

$$|M_{1}\rangle =\frac{1}{\sqrt{\kappa }}[|0\rangle +\frac{1}{\sqrt{(1-\gamma {)}^{n+m}}}|1\rangle ],\;|M_{2}\rangle =\frac{1}{\sqrt{\kappa }}[|0\rangle -\frac{1}{\sqrt{(1-\gamma {)}^{n+m}}}|1\rangle ],\;\kappa =1+\frac{1}{(1-\gamma {)}^{n+m}},$$ (29)

and the *μ* related to parameter *γ* should ensure that *O*_3_ is a semi positive operator. To determine *μ*, we need to rewrite $O_{1},O_{2}$ and *O*_3_ in matrix form

$$O_{1}=\frac{1}{\mu \kappa }\left(\begin{array}{cc} 1 & \frac{1}{\sqrt{(1-\gamma {)}^{n+m}}}\\ \frac{1}{\sqrt{(1-\gamma {)}^{n+m}}} & \frac{1}{(1-\gamma {)}^{n+m}} \end{array}\right),\;O_{2}=\frac{1}{\mu \kappa }\left(\begin{array}{cc} 1 & -\frac{1}{\sqrt{(1-\gamma {)}^{n+m}}}\\ -\frac{1}{\sqrt{(1-\gamma {)}^{n+m}}} & \frac{1}{(1-\gamma {)}^{n+m}} \end{array}\right),\;$$ (30)

$$O_{3}=\left(\begin{array}{cc} 1-\frac{2}{\mu \kappa } & 0\\ 0 & 1-\frac{2}{\mu \kappa (1-\gamma {)}^{n+m}} \end{array}\right).$$

To make *O*_3_ a positive operator, the parameter *μ* should satisfy the condition

$$\mu \geq \frac{2}{\kappa (1-\gamma {)}^{n+m}}.$$ (31)

After performing the POVM, the receiver *r*_*m*_ is able to obtain *O*_*j*_ (j=1,2) with the following probability

$$p(O_{j})=\langle Q|O_{j}|Q\rangle =\frac{1}{\mu \kappa }\;(j=1,2),$$ (32)

where $|Q\rangle =\alpha {⊗}_{j=1}^{m}|0\rangle_{r_{j}}|0\rangle_{A}+\sqrt{(1-\gamma {)}^{n+m}}\beta {⊗}_{j=1}^{m}|1\rangle_{r_{j}}|1\rangle_{A}$. According to $(\mu \kappa {)}^{-1}$ of the POVM, the receiver *r*_*m*_ can infer the state $|F\rangle_{A}$ or $|H\rangle_{A}$ of the auxiliary qubit *A*. However, based on the value $1\;-\;2(\mu \kappa {)}^{-1}$, the receiver *r*_*m*_ can obtain *O*_3_, but cannot infer the state of the auxiliary qubit *A*. Once receiver *r*_*m*_ determines the state $|F\rangle_{A}$ (or $|H\rangle_{A}$) it means he or she knows the state $|E\rangle_{m}$ (or $|G\rangle_{m}$), and then he or she applies the operation *I* (or $\sigma_{z}$) to qubit *r*_*m*_. In this way, the state of qubits $r_{1},r_{2},\cdots ,r_{m}$ becomes $|R_{m}\rangle$ with the probability $2(\mu \kappa {)}^{-1}$ and unit fidelity.

For Eq (21), we can obtain the same conclusion, so the total success probability in POVM stage is $4(\mu \kappa {)}^{-1}$, therefore, the success probability of our scheme is $4(\mu \kappa {)}^{-1}/[1+(1-\gamma {)}^{n+m}]$. Apparently, the maximum success probability of our scheme is $2(1-\gamma {)}^{n+m}/[1+(1-\gamma {)}^{n+m}]$ if $\mu =2\kappa^{-1}(1-\gamma {)}^{-(n+m)}$. Interestingly, when γ=0, the maximum success probability is 1, and $|ℋ\rangle_{n+m}$ is (n+m)-qubit GHZ state, which means that our scheme here is standard quantum teleportation of shared secret.

### 3.3 (*n*,*m*)-Type QT based on a single generalized Bell-state measurement

In the previous subsections, attaining unit fidelity in teleporting the initially shared state necessitated the use of an auxiliary qubit, followed by a transformation involving two qubits. However, we show here that the receivers can reconstruct the target state without the need for an auxiliary qubit, albeit with a non-unit probability. This is achieved by substituting Bell-state measurements with a single generalized Bell-state measurement. To initiate this process, we construct the generalized Bell-state basis as follows:

$$|\phi_{g}^{+}\rangle =\frac{1}{\sqrt{1+(1-\gamma {)}^{n+m}}}[\sqrt{(1-\gamma {)}^{n+m}}|00\rangle +|11\rangle ],\;|\phi_{g}^{-}\rangle =\frac{1}{\sqrt{1+(1-\gamma {)}^{n+m}}}[|00\rangle -\sqrt{(1-\gamma {)}^{n+m}}|11\rangle ],\;|\psi_{g}^{+}\rangle =\frac{1}{\sqrt{1+(1-\gamma {)}^{n+m}}}[\sqrt{(1-\gamma {)}^{n+m}}|01\rangle +|10\rangle ],\;$$ (33)

$$|\psi_{g}^{-}\rangle =\frac{1}{\sqrt{1+(1-\gamma {)}^{n+m}}}[|01\rangle -\sqrt{(1-\gamma {)}^{n+m}}|10\rangle ].\;$$

Using this generalized Bell-state basis, we rewrite the initial system $|S_{n}\rangle |ℋ\rangle_{n+m}$ as follows

$$|S_{n}\rangle |ℋ\rangle_{n+m}=\frac{1}{1+(1-\gamma {)}^{n+m}}\{|\phi_{g}^{+}\rangle_{s_{1}s_{1}^{\prime }}|\Phi_{n-1}^{+}\rangle_{s_{2},s_{2}^{\prime },\cdots ,s_{n},s_{n}^{\prime }}\;\;⊗\sqrt{(1-\gamma {)}^{n+m}}(\alpha {⊗}_{j=1}^{m}|0\rangle_{r_{j}}+\beta {⊗}_{j=1}^{m}|1\rangle_{r_{j}})\;\;+|\phi_{g}^{+}\rangle_{s_{1}s_{1}^{\prime }}|\Phi_{n-1}^{-}\rangle_{s_{2},s_{2}^{\prime },\cdots ,s_{n},s_{n}^{\prime }}\;\;⊗\sqrt{(1-\gamma {)}^{n+m}}(\alpha {⊗}_{j=1}^{m}|0\rangle_{r_{j}}-\beta {⊗}_{j=1}^{m}|1\rangle_{r_{j}})\;\;+|\phi_{g}^{-}\rangle_{s_{1}s_{1}^{\prime }}|\Phi_{n-1}^{+}\rangle_{s_{2},s_{2}^{\prime },\cdots ,s_{n},s_{n}^{\prime }}\;\;⊗[\alpha {⊗}_{j=1}^{m}|0\rangle_{r_{j}}-(1-\gamma {)}^{n+m}\beta {⊗}_{j=1}^{m}|1\rangle_{r_{j}}]\;\;+|\phi_{g}^{-}\rangle_{s_{1}s_{1}^{\prime }}|\Phi_{n-1}^{-}\rangle_{s_{2},s_{2}^{\prime },\cdots ,s_{n},s_{n}^{\prime }}\;\;⊗[\alpha {⊗}_{j=1}^{m}|0\rangle_{r_{j}}+(1-\gamma {)}^{n+m}\beta {⊗}_{j=1}^{m}|1\rangle_{r_{j}})\;\;+|\psi_{g}^{+}\rangle_{s_{1}s_{1}^{\prime }}|\Psi_{n-1}^{+}\rangle_{s_{2},s_{2}^{\prime },\cdots ,s_{n},s_{n}^{\prime }}\;\;⊗[(1-\gamma {)}^{n+m}\alpha {⊗}_{j=1}^{m}|1\rangle_{r_{j}}+\beta {⊗}_{j=1}^{m}|0\rangle_{r_{j}}]\;\;+|\psi_{g}^{+}\rangle_{s_{1}s_{1}^{\prime }}|\Psi_{n-1}^{-}\rangle_{s_{2},s_{2}^{\prime },\cdots ,s_{n},s_{n}^{\prime }}\;\;⊗[(1-\gamma {)}^{n+m}\alpha {⊗}_{j=1}^{m}|1\rangle_{r_{j}}-\beta {⊗}_{j=1}^{m}|0\rangle_{r_{j}})\;\;+|\psi_{g}^{-}\rangle_{s_{1}s_{1}^{\prime }}|\Psi_{n-1}^{+}\rangle_{s_{2},s_{2}^{\prime },\cdots ,s_{n},s_{n}^{\prime }}\;\;⊗\sqrt{(1-\gamma {)}^{n+m}}(\alpha {⊗}_{j=1}^{m}|1\rangle_{r_{j}}+\beta {⊗}_{j=1}^{m}|0\rangle_{r_{j}})\;\;+|\psi_{g}^{-}\rangle_{s_{1}s_{1}^{\prime }}|\Psi_{n-1}^{-}\rangle_{s_{2},s_{2}^{\prime },\cdots ,s_{n},s_{n}^{\prime }}\;\;⊗\sqrt{(1-\gamma {)}^{n+m}}(\alpha {⊗}_{j=1}^{m}|1\rangle_{r_{j}}-\beta {⊗}_{j=1}^{m}|0\rangle_{r_{j}}),$$ (34)

where $|\Phi_{n-1}^{+}\rangle$, $|\Phi_{n-1}^{-}\rangle$, $|\Psi_{n-1}^{+}\rangle$ and $|\Psi_{n-1}^{-}\rangle$ are defined in Eq (17). In this scenario, the sender *s*_1_ performs a generalized Bell-state measurement on the qubit pair $\left(s_{1},s_{1}^{\prime }\right)$. Actually, any sender within the group can perform the generalized Bell-state measurement due to the symmetry of the initial state $|S_{n}\rangle$ and the network channel $|ℋ\rangle_{n+m}$, yielding identical results. Without loss of generality, we assume the first sender is tasked with this operation, while the other senders proceed with the standard Bell-state measurements. It is clear that the result remains unchanged regardless of the order in which the joint measurements are carried out.

If the measurement result is $|\phi_{g}^{+}\rangle |\Phi_{n-1}^{\pm }\rangle$, the state available to the receivers will be $\alpha {⊗}_{j=1}^{m}|0\rangle_{r_{j}}\pm \beta {⊗}_{j=1}^{m}|1\rangle_{r_{j}}$. Similarly, if the outcome corresponds to $|\psi_{g}^{-}\rangle |\Psi_{n-1}^{\pm }\rangle$, the resulting state at the receivers’ side will be $\alpha {⊗}_{j=1}^{m}|1\rangle_{r_{j}}\pm \beta {⊗}_{j=1}^{m}|0\rangle_{r_{j}}$.

In both cases, the receivers can reconstruct the target state by implementing the appropriate local Pauli operations. As a result, the probability of successful state recovery is given by $p_{1}=2(1-\gamma {)}^{n+m}/[1+(1-\gamma {)}^{n+m}{]}^{2}$, where *p*_1_ denotes the success probability in the absence of an auxiliary qubit.

If the measurement outcome is $|\phi_{g}^{+}\rangle |\Phi_{n-1}^{\pm }\rangle$, the state at the receivers’ hands will be be $\alpha {⊗}_{j=1}^{m}|0\rangle_{r_{j}}\pm \beta {⊗}_{j=1}^{m}|1\rangle_{r_{j}}$, and when the outcome is $|\psi_{g}^{-}\rangle |\Psi_{n-1}^{\pm }\rangle$, the state will be $\alpha {⊗}_{j=1}^{m}|1\rangle_{r_{j}}\pm \beta {⊗}_{j=1}^{m}|0\rangle_{r_{j}}$. In both of these scenarios, the receivers have the capability to recover the target state by applying the appropriate local Pauli operators. Consequently, the successful probability is $p_{1}=2(1-\gamma {)}^{n+m}/[1+(1-\gamma {)}^{n+m}{]}^{2}$, where *p*_1_ means the successful probability without introducing an auxiliary qubit.

If the measurement outcome is $|\phi_{g}^{-}\rangle |\Phi_{n_{1}}^{\pm }\rangle$ or $|\psi_{g}^{+}\rangle |\Psi_{n-1}^{\pm }\rangle$, the unnormalized state will be $\alpha {⊗}_{j=1}^{m}|0\rangle_{r_{j}}\mp (1-\gamma {)}^{n+m}\beta {⊗}_{j=1}^{m}|1\rangle_{r_{j}}$ or $\left(1\;-\;\gamma {)}^{n+m}\alpha {⊗}_{j=1}^{m}|1\rangle_{r_{j}}\pm \beta {⊗}_{j=1}^{m}|0\rangle_{r_{j}}\right)$, respectively. Similar to Scheme 1 in Sect 3.1, the receivers can obtain the target state by introducing an auxiliary qubit to assist state evolution, and the corresponding general evolution is found by replacing $\sqrt{(1-\gamma {)}^{n+m}}$ with $(1\;-\;\gamma {)}^{n+m}$ in Eqs (22) and (24) respectively. Consequently, the success probability is $p_{2}=2(1-\gamma {)}^{2(n+m)}/[1+(1-\gamma {)}^{n+m}{]}^{2}$, where *p*_2_ represents the success probability with the introduction of an auxiliary qubit. Therefore, the total successful probability is $p=p_{1}+p_{2}=2(1-\gamma {)}^{n+m}/[1+(1-\gamma {)}^{n+m}]$.

## 4 (*n*,*m*)-Type QT of shared *d*-dimensional quantum secret in AD channel

In Sect 3, we discuss in detail the (2,2)-type quantum teleportation of shared 2-dimensional quantum secret in an AD channel. As its generalization, in this section, we consider the teleportation of shared *d*-dimensional quantum secret in an AD channel. Suppose that the senders $\left\{s_{1},s_{2},\cdots ,s_{n}\right\}$ have already shared a *d*-dimensional quantum secret

$$|S\rangle_{d}=\sum_{j=0}^{d-1}\alpha_{j}{⊗}_{k=1}^{n}|j\rangle_{s_{k}}$$ (35)

where $\alpha_{j}$ (j=0,1,⋯,d−1) are complex numbers with $\sum_{j=0}^{d-1}|\alpha {|}^{2}=1$. The objective is to securely teleport the confidential $|S\rangle_{d}$ to a network of interconnected receivers $\left\{r_{1},r_{2},\cdots ,r_{m}\right\}$. No complete trust is vested in any of the individuals involved, guaranteeing that confidential information remains inaccessible to any unauthorized individuals or group of senders or receivers at every stage of the process.The senders or receivers are considered to begin in a *d*-dimensional amplitude damping noisy environment, where the corresponding Kraus operators are given by [52]

$$K_{0}=|0\rangle \langle 0|+\sqrt{1-\gamma }\Sigma_{j=1}^{d-1}|j\rangle \langle j|$$ (36)

and $K_{j}=\sqrt{\gamma }|0\rangle \langle j|$ with j=1,2,⋯,d−1.

Using a similar quantum channel preparation method as in Stage 1 of Sect 2, with the GHZ state $\sum_{j=0}^{d-1}|j\rangle^{⊗\left(m+n\right)}/\sqrt{d}$, the generalized CNOT transformation GCNOT(|i⟩,|j⟩)=|i⟩|(i+j)modd⟩ and the inverse generalized CNOT transformation IGCNOT(|i⟩,|j⟩)=|i⟩|(i−j)modd⟩ [60], the senders and receivers will share the following (m+n)-qudit pure state:

$$|{ℋ}^{\prime }\rangle_{n+m}=\frac{1}{\sqrt{1+(d-1)(1-\gamma {)}^{n+m}}}[{⊗}_{i=1}^{n}|0\rangle_{s_{i}^{\prime }}{⊗}_{j=1}^{m}|0\rangle_{r_{j}}\;\;+\sqrt{(1-\gamma {)}^{n+m}}{⊗}_{i=1}^{n}|1\rangle_{s_{i}^{\prime }}{⊗}_{j=1}^{m}|1\rangle_{r_{j}}+\cdots \;\;+\sqrt{(1-\gamma {)}^{n+m}}{⊗}_{i=1}^{n}|d-1\rangle_{s_{i}^{\prime }}{⊗}_{j=1}^{m}|d-1\rangle_{r_{j}}].$$ (37)

We suggest a new implementation scheme for the teleportation of the shared secret state $|S\rangle_{d}$ through mutual collaboration as follows. Firstly, the initial composite system can be written as

$$\;|S\rangle_{d}|{ℋ}^{\prime }\rangle_{n+m}\;=\frac{1}{\sqrt{1+(d-1)(1-\gamma {)}^{n+m}}}[\sum_{j=0}^{d-1}\alpha_{j}{⊗}_{k=1}^{n}|j\rangle_{s_{k}}]\;\;⊗[\sum_{l=0}^{d-1}(1-\gamma {)}^{(n+m)\min(1,l)/2}{⊗}_{i=1}^{n}|l\rangle_{s_{i}^{\prime }}{⊗}_{j=1}^{m}|l\rangle_{r_{j}}]\;=\frac{1}{\sqrt{d+d(d-1)(1-\gamma {)}^{n+m}}}\sum_{s,t=0}^{d-1}|\Phi_{st}\rangle_{s_{1},\cdots ,s_{n},s_{1}^{\prime },\cdots ,s_{n}^{\prime }}[\alpha_{0}{⊗}_{j=1}^{m}|t\rangle_{r_{j}}\;\;+\sqrt{(1-\gamma {)}^{n+m}}\alpha_{1}\exp\{-2\pi is/d\}{⊗}_{j=1}^{m}|(1+t)modd\rangle_{r_{j}}+\cdots \;\;+\sqrt{(1-\gamma {)}^{n+m}}\alpha_{d-1}\exp\{-2(d-1)\pi is/d\}{⊗}_{j=1}^{m}|(d-1+t)modd\rangle_{r_{j}}],$$ (38)

with

$$\;|\Phi_{st}\rangle_{s_{1},\cdots ,s_{n},s_{1}^{\prime },\cdots ,s_{n}^{\prime }}\;=\frac{1}{\sqrt{d}}[{⊗}_{l=1}^{n}|0\rangle_{s_{l}}{⊗}_{l=1}^{n}|t\rangle_{s_{l}^{\prime }}+\exp\{2\pi is/d\}{⊗}_{l=1}^{n}|1\rangle_{s_{l}}{⊗}_{l=1}^{n}|(1+t)modd\rangle_{s_{l}^{\prime }}\;\;+\cdots +\exp\{2(d-1)\pi is/d\}{⊗}_{l=1}^{n}|d-1\rangle_{s_{l}}{⊗}_{l=1}^{n}|(d-1+t)modd\rangle_{s_{l}^{\prime }}],$$ (39)

where s,t=0,1,⋯,d−1.

By rearranging the order of the 2*n* qudits from $\left\{s_{1},s_{2},\cdots ,s_{n},s_{1}^{\prime },s_{2}^{\prime },\cdots ,s_{n}^{\prime }\right\}$ to $\left\{s_{1},s_{1}^{\prime },s_{2},s_{2}^{\prime },\cdots ,s_{n},s_{n}^{\prime }\right\}$, and we obtain the following (with subscripts omitted for simplicity).

$$|\Phi_{st}\rangle =\frac{1}{\sqrt{d^{n-1}}}\sum_{0\leq p_{1},p_{2},\cdots ,p_{n}\leq n,p_{1}+2p_{2}+\cdots +2^{d-2}p_{d-1}}N\{|\phi_{1t}\rangle^{p_{1}}\leq n\;\;⊗|\phi_{2t}\rangle^{p_{2}}\cdots |\phi_{(d-1)t}\rangle^{p_{d-1}}|\phi_{0t}\rangle^{\left(n-\sum_{j=1}^{d-1}p_{j}\right)}\},$$ (40)

with

$$|\phi_{st}\rangle =\frac{1}{\sqrt{d}}\sum_{j=0}^{d-1}\exp\{2\pi ijs/d\}|j\rangle |(j+t)modd\rangle ,$$ (41)

where s,t=0,1,⋯,d−1, and the *d*-dimensional Bell states $\left\{|\phi_{st}\rangle :s,t=0,1,\cdots ,d-1\right\}$ form a mutually orthogonal basis in a two-qudit *d*-dimensional system.

Secondly, the senders execute measurements on their individual qudit pairs to obtain *d*-dimensional Bell-state information, telling the results to the receivers. The combined measurement results from all transmitters produce a collective measurement outcome. In general, when a collective measurement yields the outcome $|\Phi_{st}\rangle$, it results in the disintegration of the state of the remaining qudits:

$$|\psi_{st}\rangle =\frac{1}{\sqrt{|\alpha_{0}{|}^{2}+(1-\gamma {)}^{n+m}\sum_{j=1}^{d-1}|\alpha_{j}{|}^{2}}}[\alpha_{0}{⊗}_{j=1}^{m}|t\rangle_{r_{j}}\;\;+\sqrt{(1-\gamma {)}^{n+m}}\alpha_{1}\exp\{-2\pi is/d\}{⊗}_{j=1}^{m}|(t+1)modd\rangle_{r_{j}}+\cdots \;\;+\sqrt{(1-\gamma {)}^{n+m}}\alpha_{d-1}\exp\{-2(d-1)\pi is/d\}{⊗}_{j=1}^{m}|(t+d-1)modd\rangle_{r_{j}}].$$ (42)

After receiving the measurement outcomes from the senders, each of the first *m*–1 receivers performs the following unitary transformation

U=|0⟩⟨t|+|1⟩⟨(t+1)modd|+⋯+|d−1⟩⟨(t+d−1)modd| (43)

on their respective qubits, and the final receivers executes the unitary transformation

$$U^{\prime }=|0\rangle \langle t|+\exp\{2\pi is/d\}|1\rangle \langle (t+1)modd|+\cdots \;\;+\exp\{2(d-1)\pi is/d\}|d-1\rangle \langle (t+d-1)modd|$$ (44)

on his/her qudit. Then the state $|\psi_{st}\rangle$ will change into

$$|\psi_{st}^{\prime }\rangle =(U^{d-1}U^{\prime })|\psi_{st}\rangle \;=\frac{1}{\sqrt{|\alpha_{0}{|}^{2}+(1-\gamma {)}^{n+m}\sum_{j=1}^{d-1}|\alpha_{j}{|}^{2}}}[\alpha_{0}{⊗}_{j=1}^{m}|0\rangle_{r_{j}}\;\;+\sqrt{(1-\gamma {)}^{n+m}}\alpha_{1}{⊗}_{j=1}^{m}|1\rangle_{r_{j}}+\cdots \;\;+\sqrt{(1-\gamma {)}^{n+m}}\alpha_{d-1}{⊗}_{j=1}^{m}|d-1\rangle_{r_{j}}].$$ (45)

To achieve a unified fidelity in restoring the initial state, we need to introduce an auxiliary qubit *A* with the initial state $|0\rangle_{A}$. Because the *m* qudits in the state $|\psi_{st}^{\prime }\rangle$ are symmetric, the result of any receiver holding this auxiliary qubit is the same. Without loss of generality, it is possible to assume that the final receiver *r*_*m*_ possesses the auxiliary qubit *A* and subsequently performs a collective matrix unitary transformation

$$𝒰=\left(\begin{array}{cccc} W & 0 & \cdots & 0\\ 0 & I & \cdots & 0\\ 0 & 0 & \ddots & 0\\ 0 & 0 & 0 & I \end{array}\right)$$ (46)

under the basis $\left\{|0\rangle_{r_{m}}|0\rangle_{A},|0\rangle_{r_{m}}|1\rangle_{A},|1\rangle_{r_{m}}|0\rangle_{A},|1\rangle_{r_{m}}|1\rangle_{A},\cdots ,|d-1\rangle_{r_{m}}|0\rangle_{A},|d-1\rangle_{r_{m}}|1\rangle_{A}\right\}$, where the block matrix 𝒰 includes the submatrix

$$W=\left(\begin{array}{cc} \sqrt{(1-\gamma {)}^{n+m}} & \sqrt{1-(1-\gamma {)}^{n+m}}\\ \sqrt{1-(1-\gamma {)}^{n+m}} & -\sqrt{(1-\gamma {)}^{n+m}} \end{array}\right)$$ (47)

and *I* denotes the 2×2 identity matrix. After applying the unitary transformation 𝒰, the product state $|\psi_{st}^{\prime }\rangle |0\rangle_{A}$ transforms into

$$𝒰(|\psi_{st}^{\prime }\rangle |0\rangle_{A})=\frac{\sqrt{(1-\gamma {)}^{n+m}}}{\sqrt{|\alpha_{0}{|}^{2}+(1-\gamma {)}^{n+m}\sum_{j=1}^{d-1}|\alpha_{j}{|}^{2}}}[\alpha_{0}{⊗}_{j=1}^{m}|0\rangle_{r_{j}}\;\;+\alpha_{1}{⊗}_{j=1}^{m}|1\rangle_{r_{j}}+\cdots +\alpha_{d-1}{⊗}_{j=1}^{m}|d-1\rangle_{r_{j}}]|0\rangle_{A}\;\;+\frac{\alpha_{0}\sqrt{1-(1-\gamma {)}^{n+m}}}{\sqrt{|\alpha_{0}{|}^{2}+(1-\gamma {)}^{n+m}\sum_{j=1}^{d-1}|\alpha_{j}{|}^{2}}}{⊗}_{j=1}^{m}|0\rangle_{r_{j}}|1\rangle_{A}.$$ (48)

Finally, the receiver *r*_*m*_ performs a single-qubit projective measurement on the auxiliary qubit *A* in the *Z*-basis {|0⟩,|1⟩}. If the outcome is $|0\rangle_{A}$, the receivers obtain the target state $\sum_{k=0}^{d-1}\alpha_{k}{⊗}_{j=1}^{m}|k\rangle_{r_{j}}$, which signifies a successful teleportation with fidelity 1.The success probability for this scenario is $\left(1-\gamma {)}^{n+m}/[d+d(d-1)(1-\gamma {)}^{n+m}\right]$, where the probability of obtaining the measurement result $|\Phi_{st}\rangle$ is $1/[d+d(d-1)(1-\gamma {)}^{n+m}]$, and the probability of measuring $|0\rangle_{A}$ is $(1-\gamma {)}^{n+m}$. Conversely,if the outcome is $|1\rangle_{A}$, the teleportation is failed.

According to the above discussion, we can see from Eq (38) that for any s,t∈{0,1,⋯,d−1}, the successful probability is $\left(1-\gamma {)}^{n+m}/[d+d(d-1)(1-\gamma {)}^{n+m}\right]$. Therefore, the total probability of our scheme is $d^{2}(1-\gamma {)}^{n+m}/[d+d(d-1)(1-\gamma {)}^{n+m}]$$=d(1-\gamma {)}^{n+m}/[1+(d-1)(1-\gamma {)}^{n+m}]$.

**Remark.** When *r* = 0, the state $|{ℋ}^{\prime }\rangle_{n+m}=\frac{1}{\sqrt{d}}\sum_{k=0}^{d-1}\;{⊗}_{i=1}^{n}\;|k\rangle_{s_{i}^{\prime }}\;{⊗}_{j=1}^{m}\;|k\rangle_{r_{j}}$, and the success probability is $d(1-\gamma {)}^{n+m}/[1+(d-1)(1-\gamma {)}^{n+m}]=d/[1+(d-1)]=1$, which means that our scheme is the standard scheme [49].

## 5 Discussion and conclusion

Accessible information—Any subparties cannot fully access the quantum secret during the teleportation procedures.In fact, taking Sect 3.1 as an example,suppose for simplicity that *m* = 1, and a single sender *s*_*k*_ tries to reconstruct the secret at their location using the results announced by the other senders.Once all the other senders except *s*_*k*_ execute a Bell-state measurement, the remaining state is either $|\phi^{-}\rangle_{s_{k}}[\alpha |0\rangle +\sqrt{(1-\gamma {)}^{n+m}}\beta |1\rangle {]}_{r}+|\phi^{+}\rangle_{s_{k}}[\alpha |0\rangle -\sqrt{(1-\gamma {)}^{n+m}}\beta |1\rangle {]}_{r}$ or $|\psi^{-}\rangle_{s_{k}}[\sqrt{(1-\gamma {)}^{n+m}}\alpha |1\rangle +\beta |0\rangle {]}_{r}+|\psi^{+}\rangle_{s_{k}}[\sqrt{(1-\gamma {)}^{n+m}}\alpha |1\rangle -\beta |0\rangle {]}_{r}$. By tracing out the receiver’s party, the reduced state at his or her party is either $|\alpha {|}^{2}|00\rangle \langle 00|+(1-\gamma {)}^{n+m}|\beta {|}^{2}|11\rangle \langle 11|$ or $(1-\gamma {)}^{n+m}|\alpha {|}^{2}|01\rangle \langle 01|+|\beta {|}^{2}|10\rangle \langle 10|$ unless the entire channel is under their control.Therefore, only the amplitude information is accessible to *s*_*k*_. The same applies to any subparties of senders and receivers.

It is evident that multiple distinct approaches exist for constructing generalized Bell-state basis. For example, the vectors


$$|\phi_{g}^{+}\rangle =\frac{1}{\sqrt{1+(1-\gamma {)}^{n+m}}}[|00\rangle +\sqrt{(1-\gamma {)}^{n+m}}|11\rangle ],$$

$$|\phi_{g}^{-}\rangle =\frac{1}{\sqrt{1+(1-\gamma {)}^{n+m}}}[\sqrt{(1-\gamma {)}^{n+m}}|00\rangle -|11\rangle ],$$

$$|\psi_{g}^{+}\rangle =\frac{1}{\sqrt{1+(1-\gamma {)}^{n+m}}}[|01\rangle +\sqrt{(1-\gamma {)}^{n+m}}|10\rangle ],$$

$$|\psi_{g}^{-}\rangle =\frac{1}{\sqrt{1+(1-\gamma {)}^{n+m}}}[\sqrt{(1-\gamma {)}^{n+m}}|01\rangle -|10\rangle ]$$

also form a set of mutually orthogonal generalized Bell-state bases.

It is important to note that, except for the (*n*–1) standard Bell-state measurements utilized in Sect 3.3, all other schemes involve *n* standard Bell-state measurements, which are unnecessary. In fact, a single Bell-state measurement or generalized Bell-state measurement suffices to implement each scheme. Rewrite Eq (17) as (without normalization)

$$|\Phi_{n}^{+}\rangle =|\phi^{+}\rangle \sum_{0\leq k=0mod2\leq n}N[|-\rangle^{⊗k}|+\rangle^{⊗(2n-2-k)}]\;\;+|\phi^{-}\rangle \sum_{0\leq k=1mod2\leq n}N[|-\rangle^{⊗k}|+\rangle^{⊗(2n-2-k)}],\;|\Phi_{n}^{-}\rangle =|\phi^{+}\rangle \sum_{0\leq k=1mod2\leq n}N[|-\rangle^{⊗k}|+\rangle^{⊗(2n-2-k)}]\;\;+|\phi^{-}\rangle \sum_{0\leq k=0mod2\leq n}N[|-\rangle^{⊗k}|+\rangle^{⊗(2n-2-k)}],\;|\Psi_{n}^{+}\rangle =|\psi^{+}\rangle \sum_{0\leq k=0mod2\leq n}N[|-\rangle^{⊗k}|+\rangle^{⊗(2n-2-k)}]\;\;+|\psi^{-}\rangle \sum_{0\leq k=1mod2\leq n}N[|-\rangle^{⊗k}|+\rangle^{⊗(2n-2-k)}],\;|\Psi_{n}^{-}\rangle =|\psi^{+}\rangle \sum_{0\leq k=1mod2\leq n}N[|-\rangle^{⊗k}|+\rangle^{⊗(2n-2-k)}]\;\;+|\psi^{-}\rangle \sum_{0\leq k=0mod2\leq n}N[|-\rangle^{⊗k}|+\rangle^{⊗(2n-2-k)}],\;$$ (49)

implying that carrying out one Bell-state measurement along with 2(*n*–1) *X*-basis measurements is equivalent to performing *n* Bell-state measurements (or one generalized Bell-state measurement and (*n*–1) Bell-state measurements). In this context, the *X*-basis measurement is a single-qubit projective measurement in the basis $\left\{|\pm \rangle =(|0\rangle \;\pm \;|1\rangle )/\sqrt{2}\right\}$. Since performing two single-qubit measurements is much more experimentally feasible than a two-qubit joint measurement, it appears that we should opt for the latter, where only one Bell-state (or generalized Bell-state) measurement is performed. However, only two Bell states, $|\phi^{-}\rangle$ and $|\psi^{-}\rangle$, can be unambiguously identified, meaning the success probability of a single Bell-state measurement is restricted to 1/2 [61,62]. Therefore, in Eq (17), while performing *n* Bell-state measurements, failure occurs only when the measurement result is $|\phi^{+}\rangle^{⊗n}$ or $|\psi^{+}\rangle^{⊗n}$. As a result, the success probability increases to 1–2^−*n*^, which shows that increasing *n* enhances the probability of successfully distinguishing the logical Bell states.

From the above schemes, the success probabilities of the schemes in Sects 2, 3, and 4 are


$$\frac{2(1-\gamma {)}^{4}}{1+(1-\gamma {)}^{4}},\;\frac{2(1-\gamma {)}^{n+m}}{1+(1-\gamma {)}^{n+m}},\;\frac{d(1-\gamma {)}^{n+m}}{1+(d-1)(1-\gamma {)}^{n+m}}$$

respectively, where *n* + *m*>4 and *d*>2. Through algebraic computation, we find


$$\frac{2(1-\gamma {)}^{4}}{1+(1-\gamma {)}^{4}}>\frac{2(1-\gamma {)}^{n+m}}{1+(1-\gamma {)}^{n+m}},\;\frac{2(1-\gamma {)}^{n+m}}{1+(1-\gamma {)}^{n+m}}<\frac{d(1-\gamma {)}^{n+m}}{1+(d-1)(1-\gamma {)}^{n+m}}.$$

This means that the more participants there are, the lower the success probability of the corresponding scheme; furthermore, the success probability of high-dimensional system scheme is greater than that of low-dimensional system scheme.

Finally, let us briefly discuss the security of our schemes. The security of quantum communication depends entirely on whether the entanglement used as a quantum channel, has been securely shared by the legitimate participants in advance. In other words, we need to assess the security of the entanglement resources during their distribution. By leveraging well-established and comprehensive verification strategies [63,64] used in other similar quantum tasks, malicious attacks from external sources or deceit from internal parties can be straightforwardly detected. For simplicity, we will not elaborate further here. Thus, the security of our schemes is fully guaranteed.

In summary, building on the foundations of Refs [47] and [60], we have introduced several new conclusive quantum teleportation schemes in the amplitude damping channel, enabling the teleportation of shared 2-dimensional and high-dimensional quantum secrets from an arbitrary number of senders to an arbitrary number of receivers. It is worth emphasizing that our work is not a trivial combination of existing achievements but an innovative extension tailored to the needs of practical quantum networks, with distinct core contributions that differ significantly from previous literature: specifically, our core research object is pre-shared quantum secrets, addressing the new scenario of “secondary relay transmission of shared resources" in distributed networks rather than first-time distribution or preparation of known states; in terms of interaction mode, we have broken through the limitation of “single sender to multiple receivers" in existing studies, realizing many-to-many communication (*n* senders to *m* receivers) and ensuring no subset of senders or receivers can access the secret independently, which is fundamentally different from centralized transmission modes. The proposed schemes consist of two stages: preparing pure entangled states in the amplitude damping channel and transferring shared quantum secrets between multiple parties in the network. Research findings demonstrate that, unlike all previous methods, the fidelity of each of our schemes can consistently reach 1 regardless of the strength of amplitude damping noise. Additionally, the success probabilities of the proposed schemes depend on the dimension of the quantum system, the strength of amplitude damping, and the number of participants. Specifically, the fewer the participants or the higher the dimension of the quantum system, the higher the success probability of the corresponding scheme. This unique advantage of high-dimensional scenarios (where the teleportation success probability of d-dimensional shared secrets is higher than that of low-dimensional ones under the same noise conditions) provides references for the design of high-dimensional quantum networks.These innovations collectively constitute the unique value of our work in both theoretical and practical aspects, offering a conceptual expansion of multiparty quantum communication in AD noisy environments and laying the foundation for the realization of distributed quantum communications and computations in diverse quantum networks.

## Supporting information

S1 Appendix Proof of theorem. (PDF)
