# Supplementary material for: Quantum teleportation of shared quantum secret in amplitude-damping channel
Source: PLoS One. 2025 Nov 10;20(11):e0334902. doi: 10.1371/journal.pone.0334902 (PMC12599963; doi:10.1371/journal.pone.0334902)
Supplement: S1 Appendix — (PDF) [file pone.0334902.s001.pdf]

S1 Appendix Proof of Theorem

Theorem On condition that the Bell state $|\mathcal{B}_{00}\rangle = \frac{1}{\sqrt{2}}(|00\rangle + |11\rangle)_{AA_1}$ is prepared by Alice, when she makes projective measurement on qubit A_1 in the computational basis $\{|0\rangle, |1\rangle\}$ and gets the measurement outcome $|0\rangle_{A_1}$ in (a5) of Sect. 2, Alice shares the following four-qubit pure entangled state

$$|\mathcal{H}\rangle_{ABCD} = \frac{1}{\sqrt{1+(1-\gamma)^4}}[|0000\rangle + (1-\gamma)^2|1111\rangle]_{ABCD} \tag{1}$$

with Bob, Charlie and David.

Proof After Alice transmits the qudit A_1 to Bob through the amplitude damping channel, the density matrix of the entangled state formed by the qubit pair (A, A_1) can be expressed as:

$$\begin{aligned} \rho_b = \varepsilon(\rho) &= \sum_{j=0}^{1}(I \otimes K_j) * \rho * (I \otimes K_j)^\dagger \\ &= \frac{1}{2}[|00\rangle\langle 00| + \sqrt{1-\gamma}|00\rangle\langle 11| + \sqrt{1-\gamma}|11\rangle\langle 00| \\ &\quad + (1-\gamma)|11\rangle\langle 11| + \gamma|10\rangle\langle 10|]_{AA_1}, \end{aligned} \tag{2}$$

where $\rho = |\mathcal{B}_{00}\rangle\langle\mathcal{B}_{00}|$, and ρ_b denotes the density matrix of the entangled state created by the qubit pair (A, A_1) after Bob receives qubit A_1. Then, Bob introduces auxiliary qubit B with the initial state $|0\rangle_B$ and executes the CNOT gate on qubit pairs (A_1, B), where qubit A_1 acts the control qubit and qubit B as target qubit, the density matrix of the state composed of qubit group (A, A_1, B) is written as:

$$\begin{aligned} \rho_b^* = &\frac{1}{2}[|000\rangle\langle 000| + \sqrt{1-\gamma}|000\rangle\langle 111| + \sqrt{1-\gamma}|111\rangle\langle 000| \\ &+ (1-\gamma)|111\rangle\langle 111| + \gamma|100\rangle\langle 100|]_{AA_1B}. \end{aligned} \tag{3}$$

Subsequently, Bob transmits qubit A_1 to Charlie via the amplitude damping channel. Once Charlie acquires qubit A_1, the density matrix of the entangled state consisting of the qubit group (A, A_1, B) changes to:

$$\begin{aligned} \rho_c = \varepsilon(\rho_b^*) &= \sum_{j=0}^{1}(I \otimes K_j \otimes I) * \rho_b^* * (I \otimes K_j \otimes I)^\dagger \\ &= \frac{1}{2}[|000\rangle\langle 000| + (1-\gamma)|000\rangle\langle 111| + (1-\gamma)|111\rangle\langle 000| \\ &\quad + (1-\gamma)^2|111\rangle\langle 111| + \gamma|100\rangle\langle 100| + (1-\gamma)\gamma|101\rangle\langle 101|]_{AA_1B}. \end{aligned} \tag{4}$$

When Charlie introduces an auxiliary qubit C with the initial state $|0\rangle_C$, and then carries out the CNOT gate on qubit pair (A_1, C), where qubits A_1 and C act as the control qubit and target qubit, respectively, the density matrix of four qubits (A, A_1, B, C) is represented as

$$\begin{aligned} \rho_c^* = &\frac{1}{2}[|0000\rangle\langle 0000| + (1-\gamma)|0000\rangle\langle 1111| + (1-\gamma)|1111\rangle\langle 0000| \\ &+ (1-\gamma)^2|1111\rangle\langle 1111| + \gamma(|1000\rangle\langle 1000| + \gamma(1-\gamma)(|1010\rangle\langle 1010|]_{AA_1BC}. \end{aligned} \tag{5}$$

After that, Charlie transmits qubit A_1 to David through amplitude damping channel. After David obtains the qubit A_1,the state representation of the four qubits (A, A_1, B, C) evolves into

$$\begin{aligned} \rho_d = \varepsilon(\rho_c^*) &= \sum_{j=0}^{1}(I \otimes K_j \otimes I \otimes I) * \rho_b^* * (I \otimes K_j \otimes I \otimes I)^\dagger \\ &= \frac{1}{2}[|0000\rangle\langle 0000| + \sqrt{(1-\gamma)^3}|0000\rangle\langle 1111| + \sqrt{(1-\gamma)^3}|1111\rangle\langle 0000| \\ &\quad + (1-\gamma)^3|1111\rangle\langle 111| + \gamma|1000\rangle\langle 1000| \\ &\quad + (1-\gamma)\gamma|1010\rangle\langle 1010| + (1-\gamma)^2\gamma|1011\rangle\langle 1011|]_{AA_1BC}. \end{aligned} \tag{6}$$

David introduces auxiliary qubit D and sends qubit pair (A_1, D) to a CNOT gate, where qubits A_1 and D serve as the control qubit and target qubit, respectively, the state representation of five qubits (A, A_1, B, C, D) is represented as

$$\begin{aligned}\rho_d^* = &\frac{1}{2}[|00000\rangle\langle 00000| + \sqrt{(1-\gamma)^3}|00000\rangle\langle 11111| + \sqrt{(1-\gamma)^3}|11111\rangle\langle 00000| \\ &+ (1-\gamma)^3|11111\rangle\langle 11111| + \gamma(|10000\rangle\langle 10000| \\ &+ \gamma(1-\gamma)(|10100\rangle\langle 10100| + (1-\gamma)^2\gamma|10110\rangle\langle 10110|]_{AA_1BCD}.\end{aligned} \tag{7}$$

When David sends the qubit A_1 to Alice via amplitude damping channel, the density matrix of five qubits (A, A_1, B, C, D) evolves into:

$$\begin{aligned}\rho_a = \varepsilon(\rho_d^*) &= \sum_{j=0}^{1}(I \otimes K_j \otimes I^{\otimes 3}) * \rho_b^* * (I \otimes K_j \otimes I^{\otimes 3})^\dagger \\ &= \frac{1}{2}[|00000\rangle\langle 00000| + (1-\gamma)^2|00000\rangle\langle 11111| \\ &\quad + (1-\gamma)^2|11111\rangle\langle 00000| + (1-\gamma)^4|11111\rangle\langle 11111| \\ &\quad + \gamma(|10000\rangle\langle 10000| + \gamma(1-\gamma)(|10100\rangle\langle 10100| \\ &\quad + (1-\gamma)^2\gamma|10110\rangle\langle 10110| + (1-\gamma)^3\gamma|10111\rangle\langle 10111|]_{AA_1BCD}.\end{aligned} \tag{8}$$

Finally, Alice first applies the CNOT operation to the qubit pair (A, A_1), with A as the control qubit and A_1 as the target qubit. The state representation of the entangled state composed of the qubit group (A, A_1, B, C, D) is given by:

$$\begin{aligned}\rho_a^* = &\frac{1}{2}[|00000\rangle\langle 00000| + (1-\gamma)^2|00000\rangle\langle 10111| \\ &+ (1-\gamma)^2|10111\rangle\langle 00000| + (1-\gamma)^4|10111\rangle\langle 10111| \\ &+ \gamma(|11000\rangle\langle 11000| + \gamma(1-\gamma)(|11100\rangle\langle 11100| \\ &+ (1-\gamma)^2\gamma|11110\rangle\langle 11110| + (1-\gamma)^3\gamma|11111\rangle\langle 11111|]_{AA_1BCD}.\end{aligned} \tag{9}$$

Therefore, when Alice performs a single-qubit projective measurement on qubit A_1 in the Z-basis, it will disentangle qubit A_1 from the other qubits (A, B, C, D). If her measurement result is $|0\rangle_{A_1}$, the density matrix of the four qubits (A, B, C, D) collapses to:

$$\begin{aligned}\rho_{ABCD} &= \frac{1}{2}[|0000\rangle\langle 0000| + (1-\gamma)^2|0000\rangle\langle 1111| \\ &\quad + (1-\gamma)^2|1111\rangle\langle 0000| + (1-\gamma)^4|1111\rangle\langle 1111| \quad]_{AA_1BCD} \\ &= \frac{1}{\sqrt{2}}[|0000\rangle + (1-\gamma)^2|1111\rangle]_{ABCD} \\ &\quad + \frac{1}{\sqrt{2}}[\langle 0000| + (1-\gamma)^2\langle 1111|]_{ABCD}.\end{aligned} \tag{10}$$

After renormalizing ρ_{ABCD}, Alice obtains the following pure entangled state

$$|\mathcal{H}\rangle_{ABCD} = \frac{1}{\sqrt{1+(1-\gamma)^4}}[|0000\rangle + (1-\gamma)^2|1111\rangle]_{ABCD} \tag{11}$$

with Bob, Charlie and David.
